# The prognostic value of blood pH and lactate and metformin concentrations in severe metformin-associated lactic acidosis

**DOI:** 10.1186/2050-6511-14-22

**Published:** 2013-04-12

**Authors:** Farshad Kajbaf, Jean-Daniel Lalau

**Affiliations:** 1 Service d’Endocrinologie-Nutrition, Hôpital Sud, Amiens cedex 1, F-80054, France; 2 Université de Picardie Jules Verne, Amiens, France

**Keywords:** Type 2 diabetes, Lactate, Lactic acidosis, Metformin, Prognosis

## Abstract

**Aims:**

Analysis of the prognostic values of blood pH and lactate and plasma metformin concentrations in severe metformin-associated lactic acidosis may help to resolve the following paradox: metformin provides impressive, beneficial effects but is also associated with life-threatening adverse effects.

**Research design and methods:**

On the basis of 869 pharmacovigilance reports on MALA with available data on arterial pH and lactate concentration, plasma metformin concentration and outcome, we selected cases with a pH < 7.0 and a lactate concentration >10 mmol/L. Outcomes were compared with those described for severe metformin-independent lactic acidosis.

**Results:**

Fifty-six patients met the above-mentioned criteria. The mean arterial pH and lactate values were 6.75 ± 0.17 and 23.07 ± 6.94 mmol/L, respectively. The survival rate was 53%, even with pH values as low as 6.5 and lactate and metformin concentrations as high as 35.3 mmol/L and 160 mg/L (normal < 1 mg/L), respectively. Survivors and non-survivors did not differ significantly in terms of the mean arterial pH and lactate concentration. The mean metformin concentration was higher in patients who subsequently died but this difference was due to a very high value (188 mg/L) in one patient in this group, in whom several triggering factors were combined. Sepsis, multidrug overdoses and the presence of at least two triggering factors for lactic acidosis were observed significantly more frequently in non-survivors (*p* = 0.007, 0.04, and 0.005, respectively). This contrasts with a study of metformin-independent lactic acidosis in which there were no survivors, despite less severe acidosis on average (mean pH: 6.86).

**Conclusions:**

In 56 cases of severe metformin-associated lactic acidosis, blood pH and lactate did not have prognostic value. One can reasonably rule out the extent of metformin accumulation as a prognostic factor. Ultimately, the determinants of metformin-associated lactic acidosis appear to be the nature and number of triggering factors. Strikingly, most patients survived - despite a mean pH that is incompatible with a favorable outcome under other circumstances.

## Background

We have previously shown that neither blood lactate concentration nor plasma metformin concentration was of prognostic value with respect to mortality in so-called metformin-associated lactic acidosis (MALA) [1,2]. However, given that arterial pH and blood lactate concentration may vary (from ≤ 7.34 to 6.4 and from > 5 mmol/L to 35.5 mmol/L, respectively, in our experience [2]), a focus on severe MALA is needed in order to try to better understand the paradox whereby metformin provides impressive, beneficial effects but is also associated with life-threatening adverse effects.

## Methods

Merck Serono provided us with access to its pharmacovigilance database on metformin. We systematically searched for and studied cases recorded as “metformin-associated lactic acidosis” between January 1995 and August 2010.

The database is a compilation of all cases worldwide brought to Merck Serono’s attention during the study period. Most entries are indirect, i.e. cases for which information is transmitted to Merck Serono by local or national health authorities. Other cases are documented through spontaneous declarations (from physicians, pharmacists, patients, etc.) or via the medical literature.

On the basis of these cases, the criteria for study selection were the presence of severe lactic acidosis at admission (defined as an arterial pH < 7.0 and a blood lactate concentration >10 mmol/L) and the availability of data on the plasma metformin concentration (regardless of the assay used) and survival (defined as discharge from the intensive care unit).

Comparisons between survivors and non-survivors were made using Student’s unpaired *t*-test for quantitative, demographic variables and a chi-2 test for qualitative variables. Test results with *p*-values ≤0.05 were considered to be statistically significant.

Outcomes were compared with those in Friesecke et al. recent study [3], in which cases of severe MALA were compared with cases of similarly severe metformin-independent lactic acidosis.

The present study was approved by the local investigational review board (Commission d’Evaluation Ethique des Recherches Non Interventionnelles, avis n° 101, Espace Ethique Hospitalier Amiens Picardie, Amiens, France).

## Results

Fifty-six patients (17 males and 39 females) met the selection criteria (out of a series of 869 case reports of MALA from 32 countries. Most cases (74.7%) came from Europe.

The population’s main characteristics are summarized in Table 1. The mean ± SD (range) age was 62.9 ± 12.1 (39–83) and the mean serum creatinine level was 587 ± 279 μmol/L (57–1000). The mean arterial pH was 6.75 ± 0.17 (6.28-6.99), the mean lactate concentration was 23.07 ± 6.94 mmol/L (10.9-55.7) and the mean plasma metformin concentration was 50.64 ± 42.19 mg/L (0.8-188 mg/L). The latter value may well be the highest ever reported.

**Table 1:** Characteristics of the study population (mean ± SD) [range]

	**All patients (n = 56)**	**Survivors (n = 30)**	**Non-survivors (n = 26)**	***p*-value for the survivor/non-survivor comparison**
Age	62.9 ± 12.1 [39–83]	61.7 ± 11.1 [40–81]	64.2 ± 13.1 [39–83]	0.44
Creatinine, μmol/L	587 ± 270 [57–1000]	672 ± 265 [133–1000]	508 ± 310 [57–929]	0.09
pH	6.75 ± 0.17 [6.28-6.99]	6.73 ± 0.17 [6.30-6.98]	6.79 ± 0.17 [6.28-6.99]	0.16
Lactate, mmol/L	23.07 ± 6.94 [10.9-55.7]	22.24 ± 4.72 [14–35.3]	24.0 ± 8.9 [10.9-55.7]	0.26
Plasma metformin level, mg/L	48.3 ± 39.6 [0.8-188]	39.66 ± 34.22 [0.8-160]	63.3 ± 47.4 [1.01-188]	0.04

Only 2 patients (3.5%) had a plasma metformin concentration within the therapeutic range (based on the upper limit of 2.5 mg/L recently proposed [4]). The majority of patients (73%) displayed marked metformin accumulation, with a value over 25 mg/L.

The overall survival rate in these patients was 53.6% (30 out of 56). This rate did not depend on the date during the study period (data not shown). In other words, the outcome was not more favorable because of more recent observations in survivors. Some patients survived even with pH values as low as 6.5 and lactate and metformin concentrations as high as 35.3 mmol/L and 160 mg/L (N < 1 mg/L), respectively.

**Figure 1:**
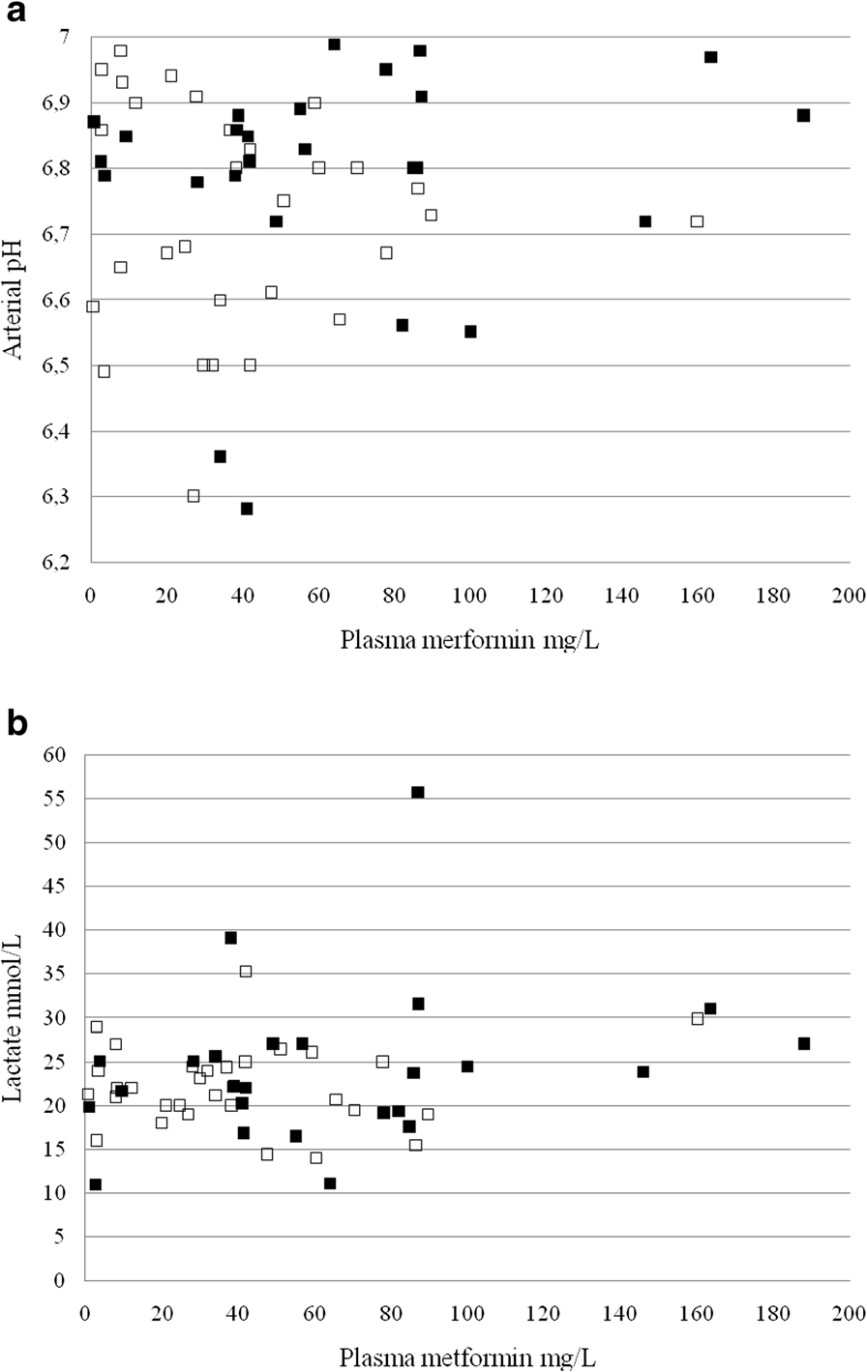
Arterial pH (a) and lactate concentrations (b) as a function of the plasma metformin concentrations and outcome in 56 patients with severe lactic acidosis (□. survivors; ■. non-survivors).

Survivors and non-survivors did not differ significantly in terms of the mean arterial pH and lactate concentration. Individual patient data for these two parameters are shown in Figure 1. The mean metformin concentration was higher in non-survivors.

The main presumed triggering factors for lactic acidosis are presented in Table 2 and are grouped into four categories: kidney failure, other organ failures, overdose and other conditions. Sepsis and multi-drug intoxication rates were significantly higher in non-survivors than in survivors (*p* = 0.007 and 0.04, respectively).

**Table 2:** Prognosis according to the presumed main triggering medical conditions (one condition per patient or more)

**Associated condition**	**Total n**	**Survivors n (%)**	**Non-survivors n (%)**	***p*-value for survivor/ non-survivor comparison**
*Kidney failure*				
Total	34	19 (55.9)	15 (44.1)	0.87
Acute failure	21	11	10	0.89
Chronic failure	7	5	2	0.43
Duration unspecified	6	3	3	1.00
*Other organ failures*				
Total	10	6	4	0.74
Liver	4	2	2	1.00
Cardiovascular system	3	3	0	0.24
Lung	3	1	2	0.59
*Overdose*				
Total	8	3	5	0.45
Metformin (alone)	4	3	1	0.61
Multidrug (including metformin)	4	0	4	0.04
*Other conditions*				
Total	13	3	10	0.02
Sepsis	6	0	6	0.007
Alcohol abuse	3	2	1	1.00
Cancer	1	0	1	0.46
Pancreatitis	1	1	0	1.00
Neurological disorder	1	0	1	0.46
Acute denutrition	1	0	1	0.46
Indetermined	3	2	1	1.00

We also analyzed the number of presumed triggering factors per patient as a function of the outcome (Table 3). The presence of only one factor was more frequent in survivors (p = 0.01) than in non-survivors; conversely, the presence of two or more combined factors appeared to be significantly more frequent in non-survivors than survivors (*p* = 0.005).

**Table 3:** Prognosis according to the number of presumed triggering medical conditions

**Number of medical conditions**	**Total n**	**Survivors n (%)**	**Non-survivors n (%)**	***p*-value for the survivor/non-survivor comparison**
No identified conditions or scarce information	3	2	1	1.00
1	38	25 (65.8)	13 (34.2)	0.01
2 or more	15	3 (20)	12 (80)	0.005

A comparison of our study results with those of Friesecke et al. [4] shows the same proportion of MALA survivors (around 50%) and the same mean pH (pH 6.75 in both studies). In contrast, Friesecke et al. did not report on any survivors in metformin-independent lactic acidosis, despite the presence of less severe acidosis (mean pH: 6.86).

## Discussion

To the best of our knowledge, this is the largest series of metformin-treated patients with severe lactic acidosis yet reported. The first striking feature is that the majority of patients (53%) survived, despite a mean arterial pH of 6.73 and a mean lactate concentration of over 20 mmol/L (22.24 mmol/L). In comparison, the mean arterial pH in our previous series of unselected MALA patients was much higher, at 7.04 (arterial lactate concentration: 15.1 mmol/L) [2].

Even though most of the patients survived (independently of age), it is important to know whether survival was related to the severity of lactic acidosis, the extent of metformin accumulation and/or the nature and number of triggering factors. A link with lactic acidosis *per se* can be ruled out, since mean pH and lactate values were similar in survivors and non-survivors. Even though the mean metformin concentration was higher in non-survivors, metformin responsibility can also be ruled out with a good degree of confidence because this concentration difference was due to a very high value (188 mg/L, N < 1 mg/L) in only one non-survivor having combined triggering factors.

Indeed, outcomes appeared to be strongly correlated with (i) the nature of the triggering factors, since some conditions were significantly more frequent in non-survivors (i.e. sepsis and multidrug intoxication, with *p* values of 0.007 and 0.04, respectively) and (ii) the number of triggering factors, since the presence of just one factor was more frequent in survivors and two or more factors were more frequently observed in non-survivors.

The fact that metformin concentrations may widely vary in MALA complicates analysis of outcomes. Indeed, metformin-treated patients do not necessarily develop lactic acidosis - even in the presence of marked metformin accumulation [5]. It is nevertheless possible to distinguish patients according to their metformin level: (i) undetectable or low, (ii) normal, (iii) slight to moderate elevation or (iv) marked elevation [6]. In our previous report, we noted that 6 of the 49 patients (12.2%) had a plasma metformin concentration at or below the upper limit of the therapeutic range [2]. The homogeneous distribution of metformin concentrations in the present series enabled us to better test for an association between metformin accumulation and outcome. Indeed, only 3.5% of the patients had a low value, whereas the great majority (almost 80%) had marked accumulation, >10 mg/L.

Lastly, we are keen to reconcile the impressively beneficial metabolic and vascular effects of metformin on one hand with the drug’s widely assumed, potentially lethal toxicity in MALA on the other – as summed up by a sentence like “metformin-associated lactic acidosis is rare but is still associated with a high mortality rate”.

Indeed, our report on a rather large series of patients with severe MALA and a death rate of almost 50% highlights how severe the complication really is. However, the problem is much more complex than that, since MALA is not a clearly defined clinical entity [7]. One way to circumvent this difficulty is thus to compare the outcome in MALA with that in metformin-independent lactic acidosis. The data from Friesecke et al. recent study (in which MALA was compared with similarly severe lactic acidosis due to other causes) showed the same proportion of survivors (around 50%) and the same mean pH (6.75) for MALA as in our (much larger) series but, strikingly, did not feature any survivors in metformin-independent lactic acidosis (despite less severe acidosis (mean pH: 6.86)) [4]. There are two possible explanations for this striking difference in survival when comparing MALA and metformin-independent lactic acidosis: (i) the presence of less severe, acidosis-triggering co-morbidities in MALA patients (because metformin accumulation is responsible for a proportion of cases of observed acidosis) and/or (ii) a protective effect of metformin (due to its vascular properties [8] and its action on the respiratory-chain complex [9]).

Whatever the underlying reason, the present report’s most remarkable finding is the unexpectedly high proportion of MALA survivors - despite an arterial pH as low as 6.5 (in 5 patients) and a lactate concentration as high as 35.3 mmol/L. However, this observation is coherent with the growing body of preclinical and clinical evidence demonstrating unexpectedly rapid recovery and survival in massive metformin accumulation and/or very severe MALA [8-13].

## Conclusions

In comparison with common forms of lactic acidosis, severe MALA is particular in that the majority of patients survive – despite a mean pH that is usually thought to be fatal. For this type of MALA patients, the outcome was related to the nature and number of triggering factors, rather than the severity of lactic acidosis or the extent of metformin accumulation.

### Key messages

•Analysis of the outcomes in severe metformin-associated lactic acidosis may help to resolve the following paradox: metformin provides beneficial effects but is also associated with life-threatening adverse effects.

•In 56 cases of severe metformin-associated lactic acidosis (pH < 7.0 and a lactate concentration >10 mmol/L), blood pH and lactate did not have prognostic value.

•The extent of metformin accumulation cannot not be considered as a prognostic factor.

•The determinants of metformin-associated lactic acidosis appeared to be the nature and number of triggering factors.

•Most patients survived despite a mean pH that is incompatible with favorable outcomes under other circumstances. Such an unexpectedly favorable outcome prompted to form a challenging hypothesis whereby metformin may be protective in severe lactic acidosis that occurs for other reasons in patients taking this drug.

## Abbreviation

MALA: Metformin-associated lactic acidosis.

## Competing interests

The authors declare that they have no competing interests.

## Authors’ contributions

FK and JDL both contributed to conception, design, acquisition of data or analysis and interpretation of data; J.D.L. was involved in drafting and revising the manuscript. Both authors read and approved the final manuscript.

## Pre-publication history

The pre-publication history for this paper can be accessed here:

http://www.biomedcentral.com/2050-6511/14/22/prepub

## References

[B1] Lalau JD Lacroix C Compagnon P Role of metformin accumulation in metformin-associated lactic acidosis Diabetes Care 1995 18 779 784 10.2337/diacare.18.6.779 7555503

[B2] Lalau JD Race J Lactic acidosis in metformin-treated patients. Prognostic value of arterial lactate levels and plasma metformin concentrations Drug Saf 1999 20 377 384 10.2165/00002018-199920040-00006 10230584

[B3] Friesecke S Abel P Roser M Outcome of severe lactic acidosis associated with metformin accumulation Crit Care 2010 14 R226 10.1186/cc9376 21171991 PMC3220003

[B4] Graham GG Punt J Arora M Clinical pharmacokinetics of metformin Clin Pharmacokinet 2011 50 81 98 10.2165/11534750-000000000-00000 21241070

[B5] Lalau JD Lemaire-Hurtel A Lacroix C Establishment of a database of metformin plasma concentrations and erythrocyte levels in normal and emergency situations Clin Drug Investig 2011 31 425 438 10.2165/11588310-000000000-00000 21401215

[B6] Lalau J Race J Metformin and lactic acidosis in diabetic humans Diabetes Obes Metab 2000 2 131 137 10.1046/j.1463-1326.2000.00053.x 11220548

[B7] Lalau JD Lactic acidosis induced by metformin Drug Saf 2010 33 727 740 10.2165/11536790-000000000-00000 20701406

[B8] Bouskela E Wiensperger N Effects of metformin on hemorrhagic shock, concentration volume and ischemia/reperfusion on nondiabetic hamsters J Vasc Med Biol 1993 4 41 46

[B9] Batandier C Guigas B Detaille D The ROS production induced by a reverse-electron flux at respiratory complex 1 is hampered by metformin J Bioenerg Biomembr 2006 38 33 42 10.1007/s10863-006-9003-8 16732470

[B10] Gjedde S Christiansen A Pedersen S Survival following a metformin overdose of 63 g: a case report Pharmacol Toxicol 2003 93 98 99 10.1034/j.1600-0773.2003.930207.x 12899672

[B11] Lalau JD Masmoudi K Unexpected recovery from prolonged hypoglycaemic coma: a protective role of metformin? Intensive Care Med 2005 3 493 15678315 10.1007/s00134-004-2540-x

[B12] Nyirenda MJ Sandeep T Grant I Severe acidosis in patients taking metformin - rapid reversal and survival despite high APACHE score Diabet Med 2006 23 432 435 10.1111/j.1464-5491.2006.01813.x 16620273

[B13] Seidowsky A Nseir S Houdret N Metformin-associated lactic acidosis: a prognosis and therapeutic study Crit Care Med 2009 37 2191 2196 10.1097/CCM.0b013e3181a02490 19487945

